# Editorial: Grazing Behavior and Welfare of Ruminants

**DOI:** 10.3389/fvets.2022.890289

**Published:** 2022-04-13

**Authors:** Luiz Carlos Pinheiro Machado Filho, Pablo Gregorini

**Affiliations:** ^1^Department of Zootechny & Rural Development, Federal University of Santa Catarina, Florianopolis, Brazil; ^2^Department of Agricultural Sciences, Lincoln University, Christchurch, Canterbury, New Zealand

**Keywords:** diet diversity, sustainable farming, social behavior, grazing patterns, lameness

The domestication and use of animals for our benefit entails responsibility for their quality of life (1). Animal welfare is a prerequisite for any ethical and sustainable animal production system to be socially defensible and acceptable (2). In nature, animals evolved in a changing environment and developed adaptive mechanisms to increase fitness (3). Cattle evolved in extensive grasslands and rangelands, in herds and families with complex social hierarchy and adapt to challenges posed by their environment through natural selection. Grazing animals face a number of challenges, including tick-borne diseases and lack of access to water and shade. Under human control, it is our responsibility to help animals to cope with such stressors and provide them a good life. This Research Topic aims to identify stressors present in pastoral husbandry systems; assess to the extent they affect health, welfare and production, and propose solutions to mitigate or overcome stressors.

To evaluate the quality of life of animals, assessing welfare conditions is necessary. A number of parameters to evaluate the welfare of grazing ruminants are proposed and are summarized by Barrell. Those parameters should include not only poor states of welfare or physiological measures, such as cortisol, endorphins, plasma serotonin, heart rate variation, etc., but also positive states. As sentient individuals, welfare assessment should include their emotional status. It is likely that to assess welfare condition in grazing ruminants, a variety of tools should be used in the methodology, rather than being reliant on a single measure (Barrell). Welfare is a more complex issue than solely the animal's condition. Welfare became a strong demand from urban societies, but they are raised on farms. Therefore, farmers have to be involved in the debate on animal welfare as part of a broader debate on environmental management, markets and social expectations. Welfare issues have to be resolved in the context of the farming system, and not considering only the experiences of the animal. More sustainable and equitable farming practices are to be built, through dialogue with all involved actors (Fisher). On this debate, pastoral systems have much to contribute. For example, overall costs of production can be reduced if heifers are raised on pasture, compared to confinement housing options (Hawkins et al.).

Grazing is a natural behavior of ruminants (4), and to offer them this opportunity is of paramount importance regarding their welfare. Providing access to alternative forms of outdoor space for ruminants appears to be less attractive for cows than pasture (Smid et al.). In natural conditions, ruminants explore and graze a diverse range of habitats. While exploring diverse habitats and swards ruminants are able to express individual grazing pattern and feed preferences, as well as personalities, with the later being regulated by social and biophysical environments, as well as the emotional state of the animal (Moreno Garcia et al.). Natural pastures offer a diverse range of forage, allowing individuals within herds to have consistent differences in grazing patterns. Such differences are best recorded by the visual observation of the foraging behavior. Ruminants have circadian rhythms and observations from dawn to midnight can represent grazing activities for the entire day (Jochims et al.).

Diet diversity is important for the animal nutrition, having an impact on animal and human health. Grazing a diverse arrangement of plants provide to ruminants different sources of primary nutrients such as protein and carbohydrates, and secondary compounds, e.g., phenolics and terpenes (Beck). A diverse pasture provides the animal a wider range and greater amount of phytonutrients, such as terpenoids, phenols, carotenoids, and anti-oxidants, than a monotonous swards, and this more than fed grain-based animals. Further, phytochemical rich diverse swards and its management improve animal health and these nutrients also benefit human health (5–7).

Besides swards' botanical composition, pasture and grazing management affect defoliation strategies by grazing ruminants, from bite features to meal behavior to daily grazing patterns. Sheep on low-intensity/high-frequency grazing strategy bitten on the “top stratum” of the plants' canopy, whereas on high-intensity/low-frequency strategy sheep mostly bitten on “grazed plants.” Selecting a diet of better nutritive value, sheep on low-intensity/high-frequency grazing had greater nutrient intake. Consequently, blood parameters of these sheep were positively associated with nutritional status and immune response to stress (Zubieta et al.), with possible positive consequences on their welfare.

Because of seasonality in pasture production and / or quality ruminants can face periods of unfulfilled nutrient demands and sometimes hunger. Among other solutions, silage, hay or other supplements offered on pasture are widely used to compensate the shortage of pasture during low season (8). As a social species, cattle and other ruminants have an internal hierarchy, where dominant animals have priority in accessing resources over subordinate ones, especially when resources are limited (9). Competition among animals then occurs, and low ranking animals have to develop strategies to access the resources. In a daily rotational pasture management system, when grain supplement was offered at the time of paddock entry, subordinate heifers could choose to graze fresh pasture, instead of competing for grain with dominant ones. On the other hand, offering supplements after 12 h of entering the paddock, resulted in a higher number of agonistic interactions and less time of grazing during grain offer (Bica). Therefore, supplementing at the time of entering the paddock reduce fights and offers subordinate heifers an opportunity to graze high-quality pasture, improving their welfare.

When proper sources of water are not offer, thirst becomes another critical challenge to grazing ruminants. Comparing the behavior and performance of grazing steers in the context of water availability in troughs or in ponds, troughs were superior with steers gaining 29% more weight (*P* ≤ 0.007) than their counterparts drinking from ponds (Bica et al.).

Identified as one of the three most common health issue affecting dairy cows (Sadiq et al.), lameness also occurs in pasture based dairy systems (10). In grazing cows, lameness can be a further problem as they have to walk more (11). Regardless of being on pasture or confined, a study showed that preventive hoof trimming was effective in reducing the prevalence of lesions (Sadiq et al.). Notwithstanding, trimmed cows that spent more time on pasture, have a lower incidence of hoof lesions (12).

Except for extensive management systems, the formation of new groups of animals and the consequent movement of animals from one group to another is a common routine in animal husbandry, including grazing ruminants. Group changing may lead to social instability and stress, implying potentially negative effects on animal welfare (Sosa et al.). As to mitigate the stressful consequences, whenever it is possible, familiar individuals should be transfered as a group and juveniles with a familiar adult. The presence of familiar adults among juveniles in a new group is likely to bring more stability and reduce aggression (13).

Grazing ruminant production systems have the potential to allow the animals to express their natural behavior, maintain health, and experience positive emotional states. The challenges ruminants face on pasture (differences in vegetation, topography, weather changes, social interactions, etc.) may also be viewed as sources leading to positive emotional states, since they present complex problems, that can be successfully solved. The diversity occurring in natural systems may improve animal welfare and prepare the animal for an efficient adaptation to environmental challenges (Villalba and Manteca).

## Author Contributions

All authors listed have made a substantial, direct, and intellectual contribution to the work and approved it for publication.

## Conflict of Interest

The authors declare that the research was conducted in the absence of any commercial or financial relationships that could be construed as a potential conflict of interest.

## Publisher's Note

All claims expressed in this article are solely those of the authors and do not necessarily represent those of their affiliated organizations, or those of the publisher, the editors and the reviewers. Any product that may be evaluated in this article, or claim that may be made by its manufacturer, is not guaranteed or endorsed by the publisher.
